# The tale of an endemic shrimp’s exceptional osmoregulation and the ancient Athalassic mangrove oasis

**DOI:** 10.1038/s41598-024-56907-4

**Published:** 2024-03-20

**Authors:** Bruno W. Giraldes, Sonia Boughattas, Fatiha M. Benslimane, Asmaa A. Althani, Christoph D. Schubart, Carla S. R. Huber, Laura R. P. Utz, Jassim A. A. Al-Khayat, Fadhil N. Sadooni, Enelise M. Amado

**Affiliations:** 1 Environmental Science Center (ESC), Qatar University, PO Box 2713, Doha, Qatar; 2 Biomedical Research Centre (BRC), Qatar University, Doha, Qatar; 3 Zoology and Evolutionary Biology, University of Regensburg, Regensburg, Germany; 4 Faculdade de Biociências, Pontifícia Universidade Católica Rio Grande do Sul (PUC-RS), Porto Alegre, Brazil; 5 Centro de Ciências Biológicas e Sociais Aplicadas (CCBSA), Universidade Estadual da Paraíba, Campus V João Pessoa (UEPB), João Pessoa, Brazil

**Keywords:** Adaptive radiation, Phylogenetics, Ecosystem ecology, Animal physiology

## Abstract

The hyperarid mangrove in the Middle East is characterised by the absence of rivers or freshwater inputs and is one of the most extreme settings of this ecosystem on Earth. Endemic to Qatar’s hyperarid mangroves, a *Palaemon* shrimp is uniquely confined to a sole mangrove site in the Arabian Gulf. Within these mangrove channels, we unveiled brine groundwater sources exceeding 70 ppt salinity, contrasting the local marine standard of 42 ppt. Concurrently, a mysid species typically linked to salt pans and groundwater coexists. Stable isotopic analysis implied the existence of a predator–prey dynamic between this mysid species and the studied shrimp. Then, investigating the endemic shrimp’s adaptation to extreme salinity, we conducted osmolarity experiments and phylogenetic studies. Our findings demonstrate that this shrimp transitions from hypo- to hyper-osmoregulation, tolerating salinities from 18 to 68 ppt—an unprecedented osmoregulatory capacity among caridean shrimps. This speciation pattern likely arises from the species osmolarity adaptation, as suggested for other *Palaemon* congeners. Phylogenetic analysis of the studied *Palaemon*, along with the mangrove’s geological history, suggests a profound evolutionary interplay between the ecosystem and the shrimp since the Eocene. This study proposes the hyperarid mangrove enclave as an Athalassic mangrove oasis—a distinctive, isolated ecosystem within the desert landscape.

## Introduction

Mangroves are one of the most crucial ecosystems on Earth, possessing significant economic, ecological, and social importance^1–3^. They represent the primary biome found in tropical estuaries, acting as a boundary between freshwater from rivers and saltwater from oceans^3,4^. However, in subtropical regions, hyperarid mangroves are characterised by the absence of rivers^4–6^ and the expected lack of freshwater inputs. Despite this, the daily tidal current in these hyperarid mangroves creates channels resembling rivers, along with the development of riparian zones along their margins. These riparian zones possess unique features and offer distinct habitat complexity within the mangrove ecosystem^7–10^. Based on the Aridity Index (AI), this mangrove is classified as hyperarid, with a ratio below 0.05 between mean annual precipitation and evapotranspiration^4^. A forest ecosystem in a desert environment is characterised by low precipitation, high temperatures, high evaporation rates, and the absence of freshwater inputs, ultimately leading to a hyper-saline mangrove ecosystem^4,5,11^.

The hyperarid mangrove found in the Middle East is one of the largest and most extreme settings of this ecosystem on Earth, with dense forests located along the Red Sea and Arabian Persian Gulf. A particularly unique mangrove setting exists in the southwestern province of the Arabian Persian Gulf ecoregion, isolated by hyperthermic and hypersaline biogeographic barriers^4,5^. This province is recognised as an extreme ecoregion, with mangroves experiencing intense temperatures and salinities. In the supratidal zone, tidal pools come into contact with the desert ecosystem, resulting in air temperatures of up to 56 °C, water temperatures of 48 °C, and salinities of 74 ppt during summer^4,5,12^. Within this hyperarid mangrove province, high endemism and low species diversity are observed^5,12,13^ housing only species adapted to survive in these extreme conditions. Including the existence of only *Avicennia marina* (Forssk.) Vierh. as a habitat builder^4,6,14^, the occurrence of only one bioturbating crab species per tidal zone^5^, and recently described species apparently endemic to the mangroves in this ecoregion, such as sponges^15^, Alpheid shrimps (in Publishing process), and the palaemonid *Palaemon khori* De Grave & Al-Maslamani, 2006^16^*,* which has strong ecological ties to the mangrove ecosystem. The resident fauna within the mangrove relies heavily on local resources^17^. It is worth noting that the East coast of this Gulf represents another distinct province characterised by constant water influx from the Indian Ocean and presenting a higher diversity of species originally from the Indian Ocean occupying the niches in their mangrove ecosystem^14,18,19^.

The shrimp *P. khori* was described in 2006^16^ and has not been documented in any other hyperarid mangroves within the Gulf or Red Sea^17,20–23^. This species exhibits high abundance, forming dense populations within the riparian zones of channels in the hyperarid mangrove located in the southwestern regions of the Gulf^8,16^. It coexists as a permanent resident in the riparian zone with only a few other species, including the endemic fish *Aphaniops dispar* (Rüppell, 1829)^8^. The endemic *P. khori* has been observed across all tidal zones within this mangrove setting, including tidal pools in the high intertidal levels during summer when salinity and temperature levels are elevated. These findings suggest that this shrimp must possess highly efficient osmotic mechanisms to survive under such extreme conditions.

In terms of crustacean osmolarity, fully marine species are generally stenohaline osmoconformers, relying on a constant salinity level in their environment. In contrast, estuarine and freshwater crustaceans, both stenohaline and euryhaline, are active osmoregulators capable of tolerating certain fluctuations in salinity^24–27^. Decapods are keystone species in marine ecosystems and can be considered bioindicators of salinity levels, where their niche and site occurrences can be based on specific salinity-induced physiological constraints associated with their osmoregulation pattern^28^. The regulation and toleration of particular salinity rates of some estuarine palaemonid shrimps are related to evolutionary adaptation for colonising specific environmental settings^25,29–31^. *Palaemon* are recorded inhabiting mangroves, estuaries, fresh water and brackish water and are constantly recorded in mangrove environments^29,32,33^. They are recognised as efficient osmoregulators, adapted to different salinity levels in estuaries^29,34^. These species often display niche specificity, and consistently exhibit speciation and niche separation based on salinity levels, with some congeners adapted to inhabit areas with lower salinity upstream in rivers, while others thrive in estuarine areas near the sea^29,32–34^.

In this study, we utilise *P. khori* as a model species to enhance our understanding of the hypersaline mangrove ecosystem in the southwestern province of the Arabian-Persian Gulf. Our objectives include evaluating the salinity tolerance and osmoregulation of this endemic shrimp, investigating its phylogeny and phylogeographic relationship with other congeners, exploring its trophic interactions with other associated species, and describing some ecological information. Additionally, the study incorporates geological data to support the comprehension of the unique hyperarid mangrove environment, its existence, and its isolation.

## Results

### Ecological relations

**Figure 1:**
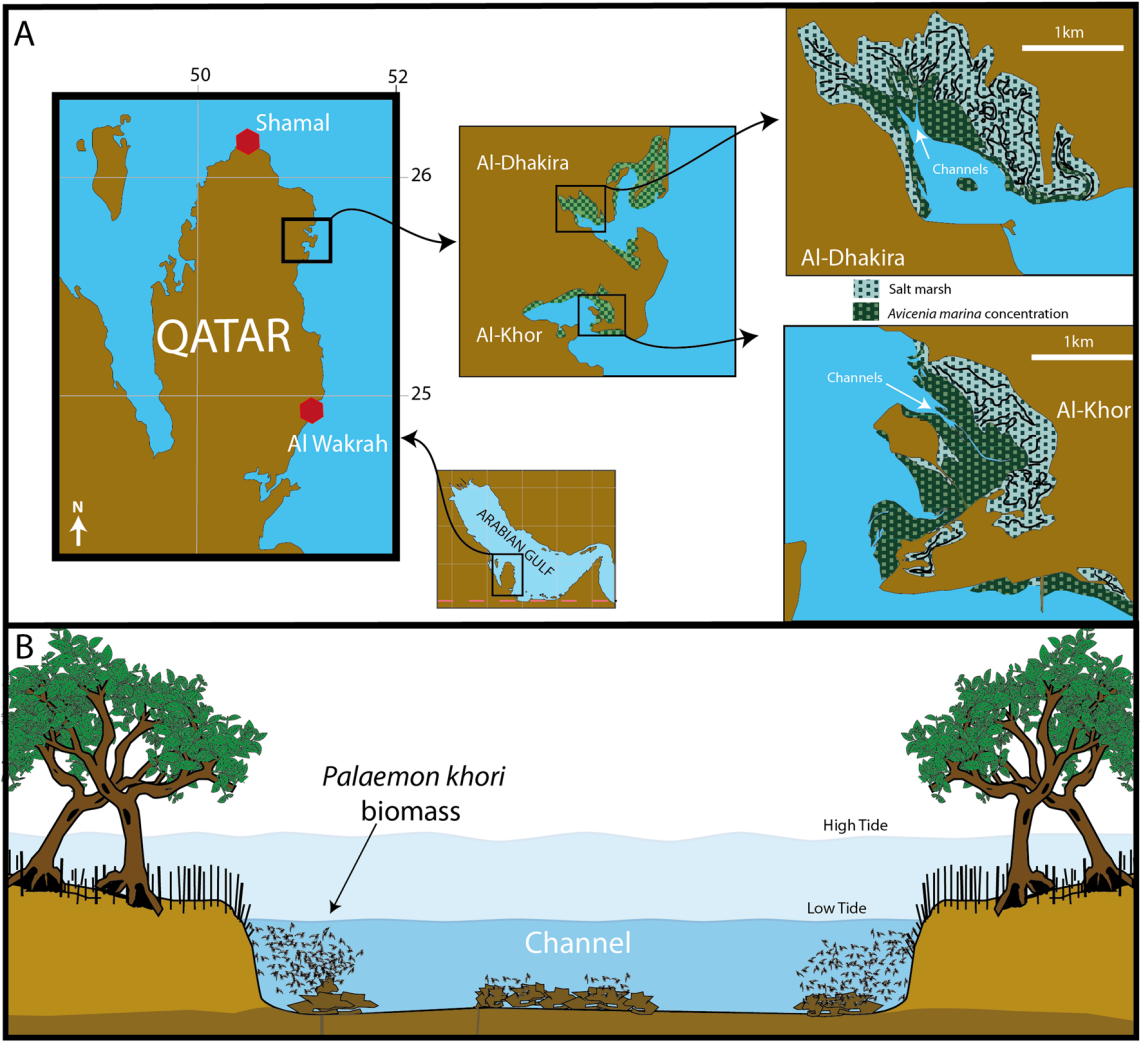
The studied mangrove, highlighting (**A**) the studied mangrove areas in Qatar, Arabian Gulf, with emphasis on the complex Al-Dhakira-Al-Khor; (**B**) the riparian zone in a channel profile with the dense biomasses of *Palaemon khori*.

Several groundwater outflows were recorded among the rocks in the channels in the Al-Dakhira & Al-khor mangrove complex (Fig. 1). The water from those groundwater outflows was colder (average of 28 °C) and had higher salinity (average of 59.8 ppt). In comparison, the seawater influx from the sea recorded a temperature of 36 °C (during summer) and a salinity of 42 ppt. In the supratidal zone, the water temperature reached 45 °C and the salinity overreached 70 ppt. Demonstrating the viability of the methodology for searching for groundwater outflow and illustrating the higher salinity on the recorded groundwater outflow and in the supratidal zone.

Several demersal shoals of *P. khori* with hundreds of individuals were recorded in the riparian zone on the channels at Al-Khor & Al-Dakhyra mangrove complex (Fig. 1), some on the substrate (mangrove roots, rocks etc.) but the majority remaining in the water mass. This shrimp's biomass was recorded on the entire mangrove, with reduced biomass closer to the channel opening with the sea; and small biomasses on the extremely hot and saline tide pools in supratidal areas. These shrimps were not observed on the beaches next to the mangrove or in the planted mangrove in Al-Wakrah (Fig. 1). In the scattered mangrove in Shamal, a small patch of supposedly *P. khori* was observed in a rocky tide pool next to the fragmented mangroves. Representatives of these shrimps in Shamal were used in the genetic analysis, and they were recorded as a different species (see genetic studies below).

**Figure 2:**
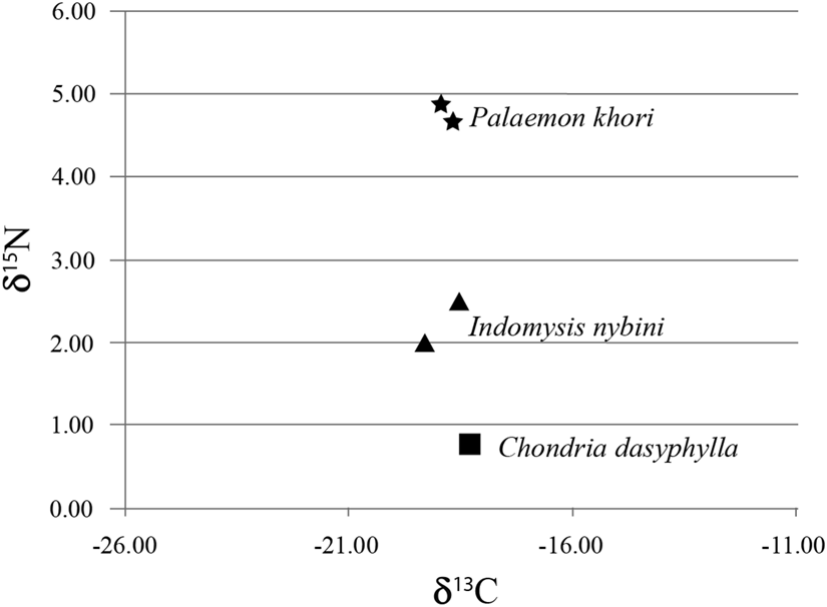
The recorded isotopes results of carbon (δ^13^C) and nitrogen (δ^15^N) of the material collected in a riparian zone, in the studied mangrove, aligning the red algae with the mysid and the Palaemonid shrimp.

In the type locality of *P. khori*, some mysids from the species *Indomysis nybini* Biju & Panampunnayil, 2010, were observed and collected in the planktonic samplings. The mysid biomass was recorded swimming close to the water surface and next to the substrate in the riparian zone, just above the biomass of *P. khori* (Fig. 1B)*.* The red algae *Chondria dasyphylla* (Woodward) C. Agardh, 1817 was recorded covering the substrate in the roots where the mysid and the studied palaemonid were collected. In their trophic relations, we recorded a possible predatory association between *P. khori* and *I. nybini* (Fig. 2)*,* where both were aligned in the similar carbon (δ^13^C) marks with − 18.81  ±  0.12‰ and − 19.22  ±  0.68‰, respectively, and the red algae on the roots in the riparian habitat with − 18.30‰; and the nitrogen (δ^15^N) values suggesting a predatory sequence with 4.7  ±  0.1‰ and 2.25  ±  0.27‰, respectively, and the red algae with 0.81‰.

### Osmolarity

**Figure 3:**
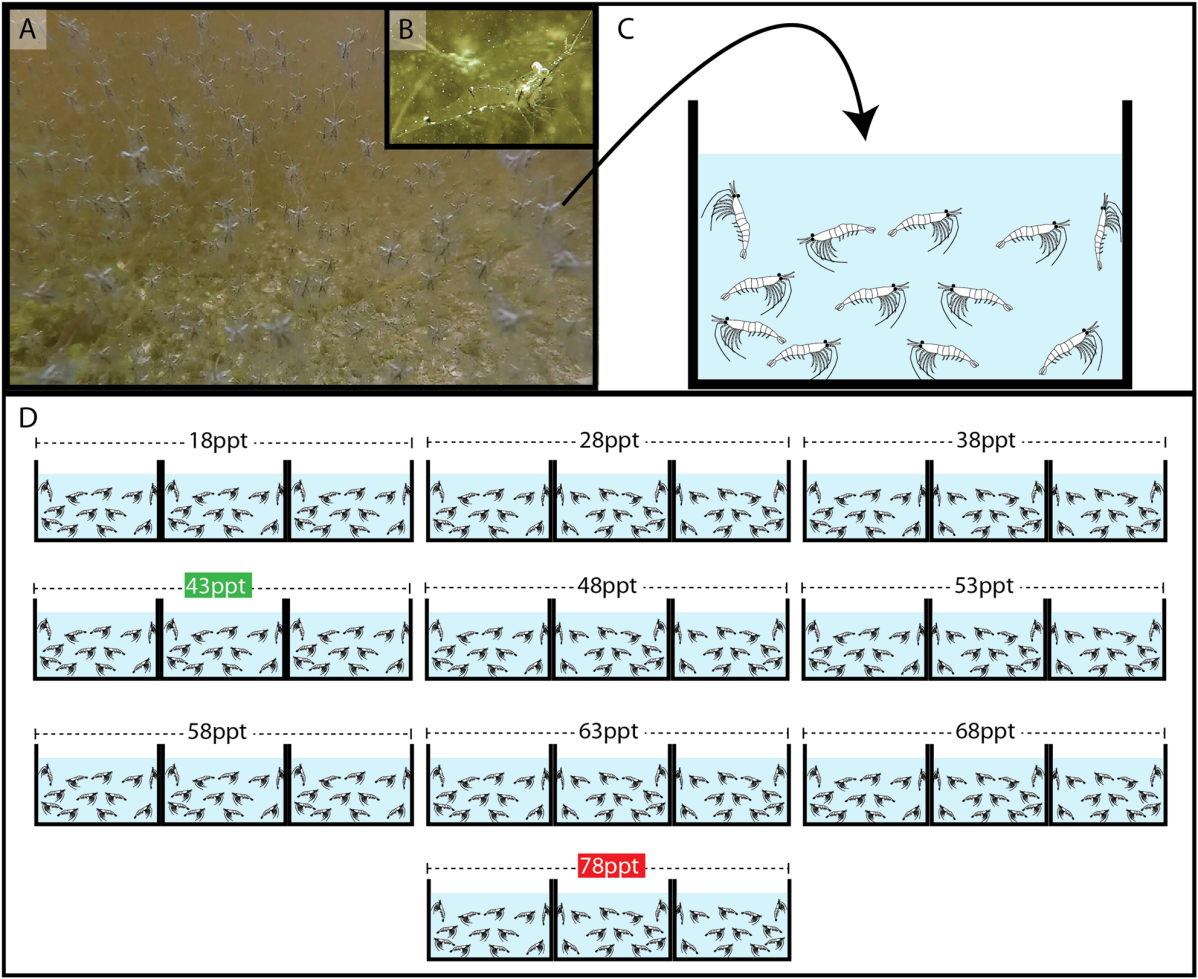
Salinity tolerance experiment. (**A**) the dense biomass of *Palaemon khori* in the field (**B**) a close-up of this palaemonid. (**C**) representation of transposed shrimps to the aquariums. (**D**) the experiment setup with ten salinities levels.

**Figure 4:**
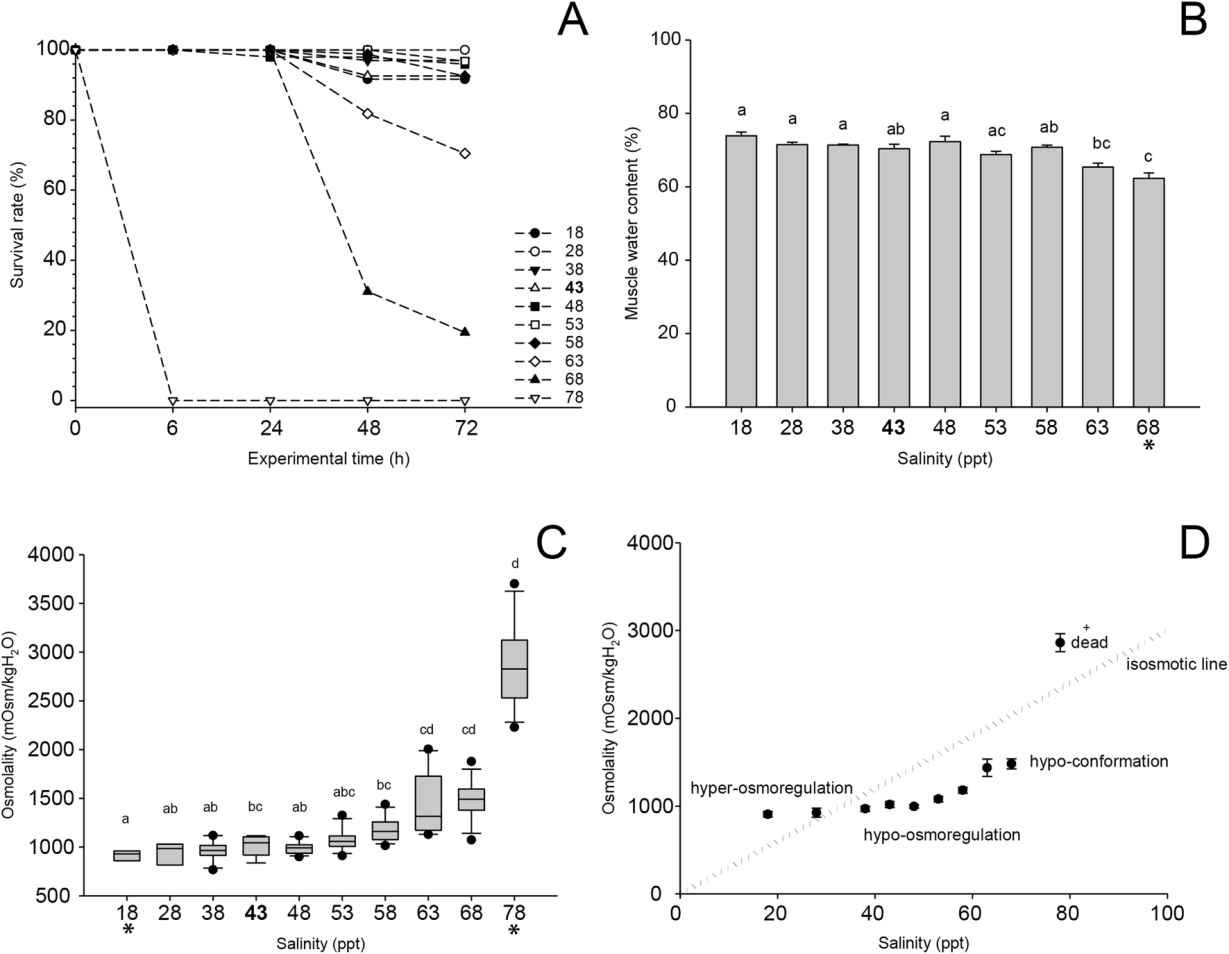
Salinity tolerance experiments with *P. khori*, illustrating (**A**) survival timeline per treatment, (**B**) muscle hydration per treatment, (**C**) hemolymph osmolarity per treatment, and (**D**) osmotic pattern compared with the isosmotic line. Bold ‘43’ represents the local marine salinity. In (**B**) and (**C**), treatments sharing the same letter are not significantly different, while treatments with different letters are statistically significant. *Highlights treatments significantly different from the salinity 43 treatment (Kruskal–Wallis one-way ANOVA, p < 0.05, n  =  8–10).

After the laboratory experiments exposing the shrimps to different salinity (Fig. 3), it was recorded that *P. khori* tolerated salinity levels ranging from 18 to 68 ppt (Fig. 4A). No mortality was observed at a salinity of 28 ppt, and only a few mortalities were recorded after 24 or 48 h at salinities of 18, 38, 43, 48, 53, and 58 ppt, resulting in a survival rate exceeding 90%. However, the high tolerance of *P. khori* to salinities of 63 and 68 ppt persisted only for up to 48 h, after which mortality began to occur, resulting in a 70% survival rate at a salinity of 63 ppt and a mere 20% survival rate at a salinity of 68 ppt. At a salinity of 78 ppt, 100% mortality was observed within the first 6 h (used for osmolarity measurements).

The surviving shrimps tolerate salinity variation exhibiting regulatory capacity of tissue hydration and haemolymph osmolality. Analysis of muscle water content (Fig. 4B) revealed consistent maintenance of muscle hydration of approximately 70% across salinities ranging from 18 to 58 ppt. For instance, shrimps exhibited a muscle water content of 70.4  ±  1.2% at a salinity of 43 ppt, which closely resembles the natural sea salinity in the region. However, at salinity 63 ppt, muscle started to lose water (65.3  ±  1%), and a significant decrease in muscle water content occurred at salinity 68 ppt (62.3  ±  1.4%). Regarding the osmoregulatory capacity  (Fig. 4C and D), the inner body solute was regulated, and shrimps demonstrated a visible osmotic regulation from 18 to 68 ppt. The haemolymph osmolality was maintained around 1000 mOsm/kg H_2_O across salinities ranging from 18 to 58 ppt (e.g., 1017.1  ±  34.2 mOsm/kg H_2_O at a salinity of 43 ppt). A significant increase in haemolymph osmolality was observed at salinities 63 and 68 ppt (1436  ±  98.4 and 1480.9  ±  57.2 mOsm/kg H_2_O, respectively). However, the observed osmolality remained below the predicted osmolality for these salinities (1890 and 2040 mOsm/kg H_2_O, respectively). Shrimps exhibit active hyper-osmoregulation at salinities 18 and 28 ppt, followed by hypo-osmoregulation at salinities ranging from 38 to 68. Furthermore, a tendency of hypo-conformation was observed at salinities 63 and 68 ppt until the mortality of 78 ppt, when osmotic capacity was lost.

### Phylogeny and phylogeography

Considering that no shrimps were recorded on the planted mangroves in Al-Wakrah and only representatives on a rocky tidal pool on Shamal were recorded. In the genetic comparisons, we used the representatives from this species' original type locality and the representative from Shamal, called here as *P.* aff. *khori*.

For the 16S gene, the approximate 570 bp region was successfully amplified and unambiguously sequenced. Over the 538 bp trimmed sequence, one SNP alteration was observed between the two Qatari isolates at the level of the 264th nucleotide with substitution of G (Accession number Isolate *P. khori*: OQ421809) by C (Accession number Isolate *P.* aff. *khori*: OQ421808). Both nucleotides are complementary with strong interactions.

**Figure 5:**
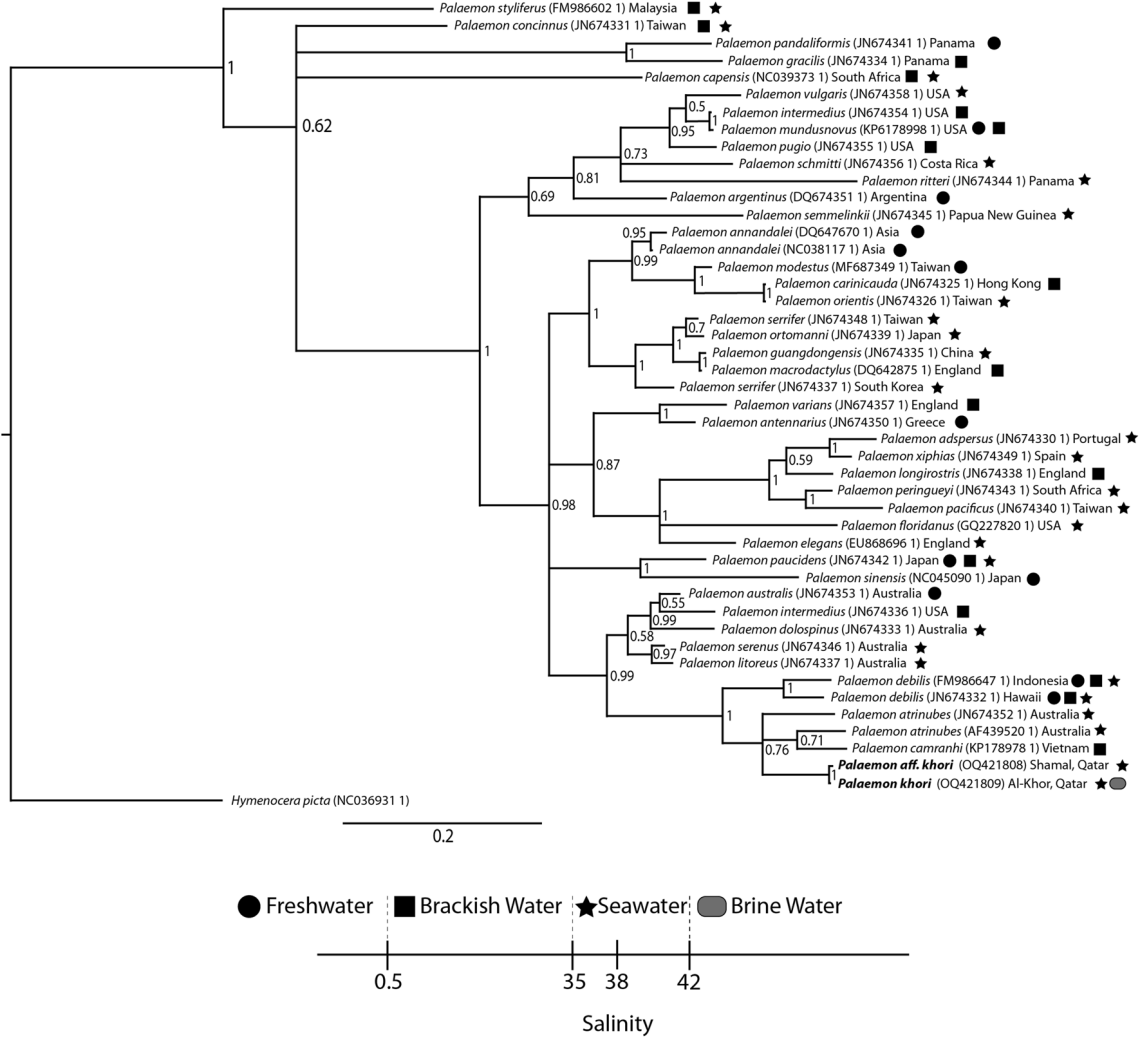
Topology of Bayesian Interference tree of different Palaemonidae identified from different geographic zones based on 16S DNA sequence data. Numbers next to the nodes represent posterior probabilities. Probabilities < 50% are not shown. The location and main habitat regarding the salinity 33 of each species are also detailed.

We aligned our isolate sequences with representative sequences from *Palaemon* Clade species reported from different regions^34,35^. The 16S dataset consisted of 47 sequences, with *Hymenocera picta* Dana, 1852 sequence used as a phylogenetic outgroup. The Bayesian tree topology advances supported genetic clustering according to the geographic distribution of the species. Indeed, *P. khori* was revealed to be clustering very closely with the Indo-Pacific *P. camranhi* (Nguyên, 1997), the Australian *P. atrinubes* (Bray, 1976) and *P*. *debilis* Dana, 1852 specimens. To a wider extent, *P. khori* is strongly supported (0.99) grouping with other different Australian species [*P. litoreus* (McCulloch, 1909); *P. serenus* (Heller, 1862); *P. dolospinus* Walker & Poore, 2003; *P. intermedius* (Stimpson, 1860); *P. australis* (Dakin, 1915)] on a separate clade different from the rest of the investigated Palaemonidae (Fig. 5).

Contrary to the 16S gene, wide sequence heterogeneity was observed within the COI gene between the two isolates of *P. khori* sampled from two different geographic locations. Different SNPs were observed along the trimmed sequences. Moreover, sequence deletion of a 30 bp region is observed within the Isolate *P.* aff. *khori* comparing the Isolate *P. khori* sequence (Accession number: OQ422167).

**Figure 6:**
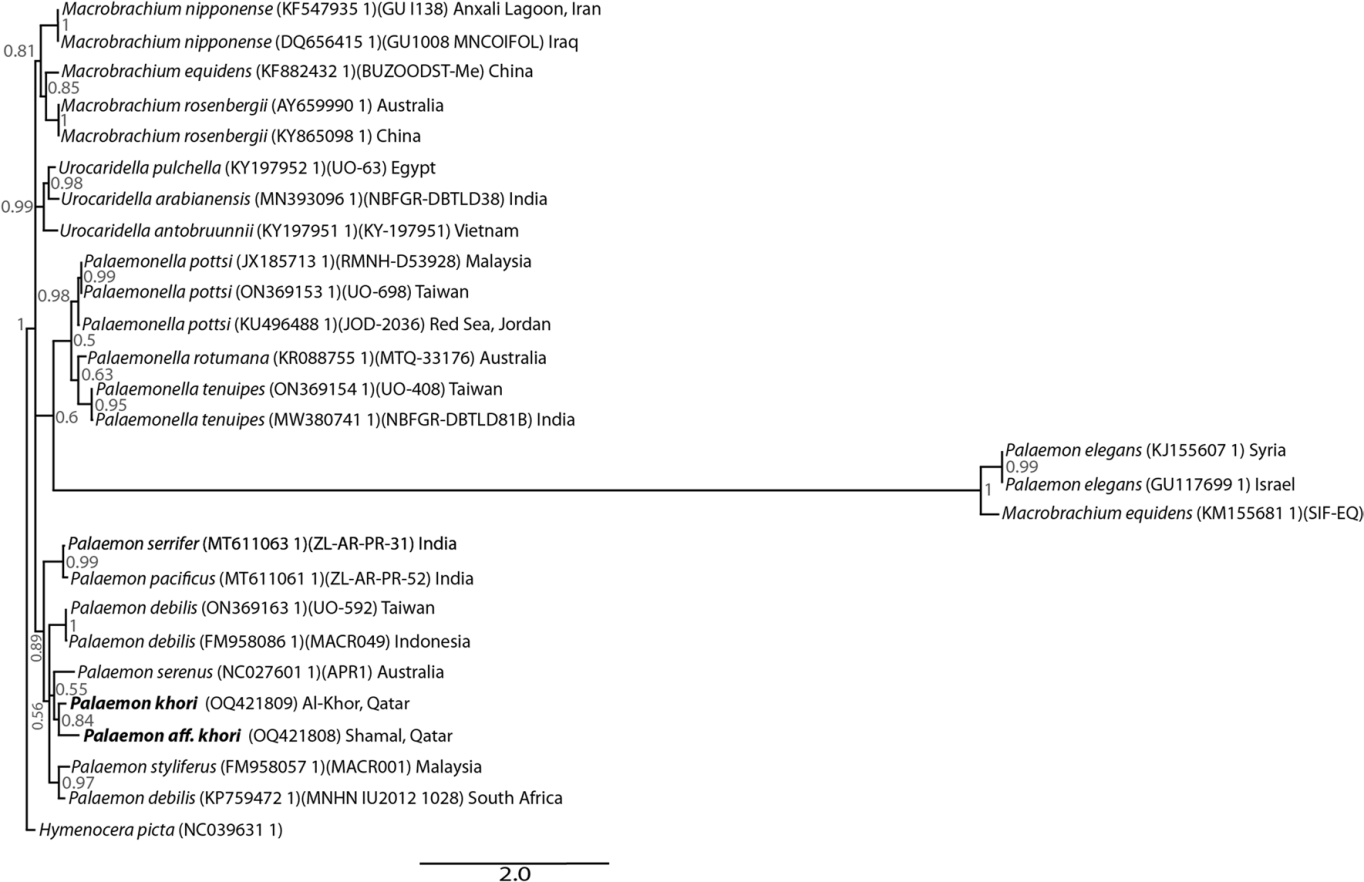
Topology of Bayesian Interference tree of different Palaemonidae identified from neighbouring geographic zones based on COI DNA sequence data. Numbers next to the nodes represent posterior probabilities. Probabilities < 50% are not shown.

Within the phylogenetic analysis, the earlier closely related *P. camranhi* and *P. atrinubes* were not included as no genetic data are available targeting their COI gene. Our sequences were then aligned with the reported species from the Indo-Pacific region as well as from Australia. Moreover, sequences of species identified in the neighbouring countries of Qatar were also included. The COI dataset included 27 sequences in total, with *Hymenocera picta* sequence used as a phylogenetic outgroup. According to the topology pf Bayesian Interference tree, *P. khori* is not related to the species identified within the Arabian-Persian Gulf [*Macrobrachium nipponense* (De Haan, 1849)*, P. elegans* Rathke, 1836] nor those reported from the Red Sea region [*P. concinnus* Dana, 1852*; P. pacificus* (Stimpson, 1860)*; Palaemonella rotumana* (Borradaile, 1898)*; P. tenuipes* Dana, 1852*; Urocaridella cyrtorhyncha* (Fujino & Miyake, 1969)*, U. pulchella* Yokes & Galil, 2006]. Indeed, the results demonstrated that the species that inhabits the intertidal zone of Qatar strongly cluster with the Australian marine *P. serenus* and the *P. debilis* identified within the Indo-Pacific (Fig. 6).

**Figure 7:**
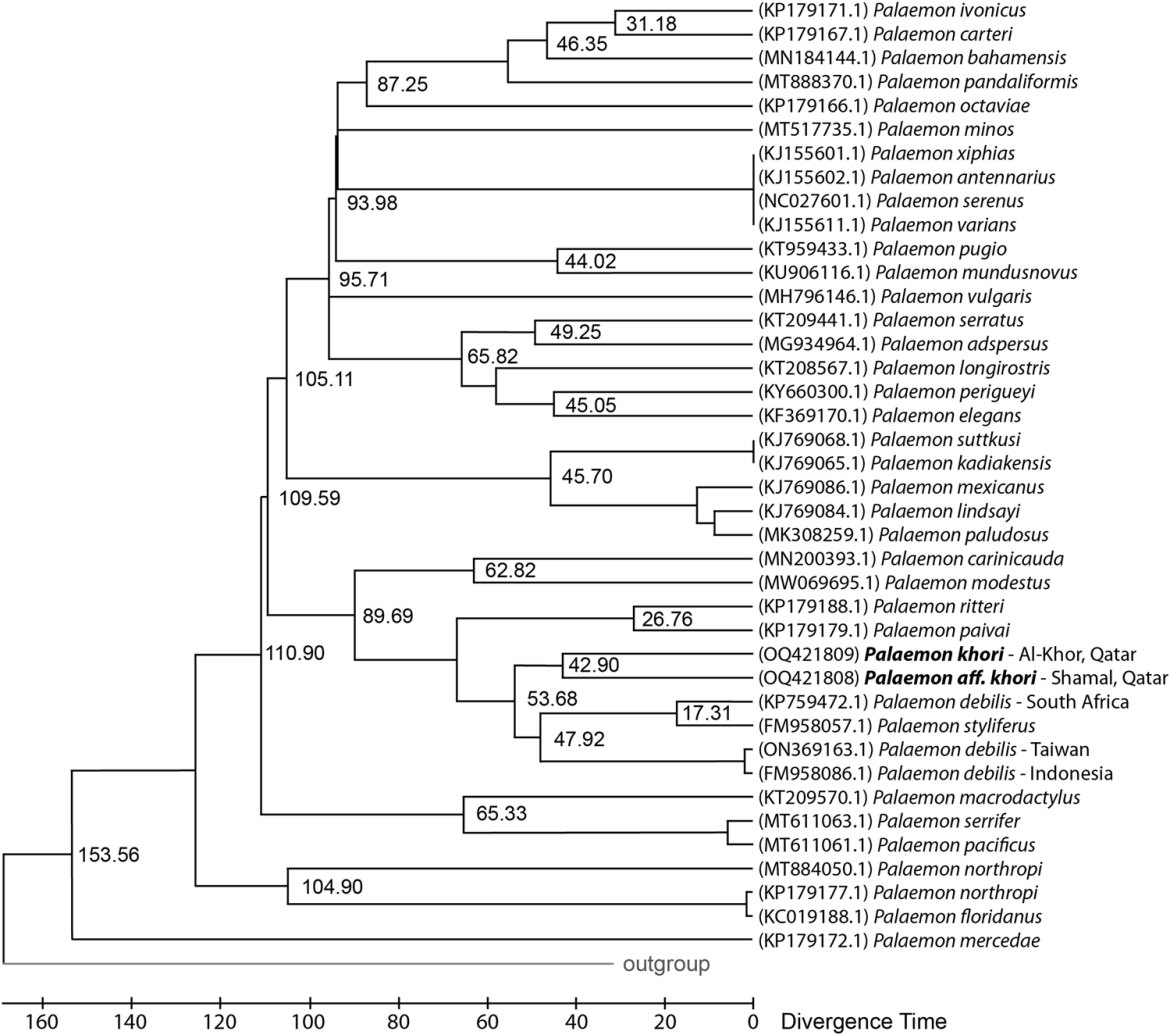
A timetree based on the Palaemonidae dataset targeting COI markers inferred by applying the RealTime approach using the ML method and the TN  +  G substitution model.

The time tree analysis included 37 representative *Palaemon* species, with *P. debilis* and *Alpheus bahamensis* Rankin, 1898 species used to set the time boundaries. The Timetree topology supports that the complex *P. khori*, *P. debilis* and *P. styliferus* emerged 53.68 MYA ago (Fig. 7) with *P. debilis* surfacing before *P. khori* (47.92 vs 42.90 MYA). The separation between *P. khori* and *P.* aff*. khori* occurred on (42.9 MYA). However, the speciation of *P. debilis* outside of the Indo-Pacific area (South Africa) occurred more recently (17.31 MYA).

## Discussion

This study has demonstrated the existence of brine groundwaters in the mangrove channels, with a salinity average (59.8) higher than the sea in the region (42). Suggesting that the studied hyperarid mangrove is linked to this brine groundwater outflow. Highlighting that a groundwater correlation was also recorded with the presence of another mangrove setting in the world^36^. The lower recorded temperature in the brine groundwater outflow also demonstrates a thermic influence of this groundwater on this ecosystem as the temperature average of this brine groundwater was 28 °C, while the temperature in the sea was 36 °C. These results suggest that the salinity and temperatures recorded in these areas are not only based on the seawater input, and this groundwater association might be linked to the evolutionary history of this mangrove setting. A better understanding of the influence of this groundwater on the mangrove ecosystem must be addressed in further studies.

Curiously, *I. nybini* was first described as inhabiting brine water in salt pans with salinity between 10 to 80^37^ and is recorded as associated with groundwater systems in the Arabian Gulf^38^. In several regions in the world and commonly associated with desertic areas, Athalassic lakes are formed by saline groundwater, most thalassic water with salinity between 35 to 50 and presenting specific Athalassic fauna physiologically adapted to the high salinity^39–41^. Considering the recorded association of the studied mangrove with the brine groundwater, the association of *I. nybini* with the groundwater in this desert region*,* and its trophic relationships with the endemic shrimp *P. khori,* it is possible to name this ancient hyperarid forest as an Athalassic mangrove oasis.

In conclusion, *P. khori* is very abundant but occurs only in a mangrove complex setting. Not recorded in other mangroves in this study, even with decapod experts searching for them in other mangrove settings in the Gulf (Reza Naderloo *per.com*.)^20–23^. Within a semi-enclosed sea with a water residence period of >  3 years and an intense tidal circulation^42^, it was expected that this species would inhabit all mangroves in the Gulf due to the natural larval dispersion of marine invertebrates. Indeed, the recorded osmolarity power would support the widespread capacity of this species to inhabit different environments. The results of this study suggest that the trophic linkage with groundwater might be an answer and that the ecology of its larval stage might clarify the explanation regarding why it remains isolated in the same mangrove setting in the southwestern region of the Gulf. The results also support the proposed isolation of the southwestern region in the Gulf^5^ as a biogeographic ecoregion.

The isotope results associate the red algae *C. dasyphylla*, with *I. nybini* and *P. khori* in a probable trophic relation. Previous studies included *P. khori* among carnivores and recorded this palaemonid as presenting the same isotope carbon and nitrogen values recorded in this study^17,43^. Demonstrating a diet pattern of *P. khori* in the studied area. A diet pattern that might support the biodiversity retention in the studied mangrove, as dietary information supports that resident fauna within arid mangrove systems mainly depends on localised retention with a smaller contribution from inwelling sources from peripheral ecosystems^17,43^. Considering the proposed zooplanktonic diet of *I. nybini*^37^, they may feed on the epifauna associated with the recorded red algae, and further study can clarify these trophic relations. The presence of the Arabic fish *A. dispar* sharing the niche with *P. khori* was previously described^8^ and considering that a large constituent of the *A. dispar* diet is plankton^44^ and that this fish was also considered as carnivorous with nitrogen isotope with values from 4.5 to 5.5‰^43^ close to the recorded nitrogen values of *P. khori* it is probable that they compete for the similar planktonic resources. Raising the importance of understanding the planktonic dynamics in the studied mangrove, including the larval stages of *P. khori* that remain morphologically undescribed. In addition, the larval behaviour of *P. khori* also remains ecologically undescribed. Considering the absence of this species in other mangroves, as recorded in this study, it is possible to speculate if this larval stage might be demersal and avoid exiting from the mangrove complex using vertical displacement, as described for other demersal meroplankton in mangrove^45^ but contrasting with other palaemonid larvae that present planktonic behaviour.

Our results on salinity tolerance demonstrated that *P. khori* exhibits remarkable osmoregulatory ability, displaying a shift in osmoregulatory pattern from hyper-osmoregulation in low salinities to hypo-osmoregulation in higher salinities, within the range of 18 to 68 ppt. However, a physiological limit was reached at salinity 78 ppt, resulting in 100% mortality within the first hours of the experiment. Versatility in the osmoregulatory pattern is a characteristic feature observed in some intertidal/estuarine palaemonid shrimps^29,46^. In palaemonid shrimps, the regulation efficiency and toleration of specific rates of salinity are related to their evolutionary adaptation for inhabiting specific environmental settings^25,29–31^. Their niche and site occurrences are based on specific salinity-induced physiological constraints associated with their osmoregulation pattern^28^. Indeed, these shrimps are considered bioindicators of salinity levels, with marked niche separation per congener according to the saline levels in freshwater, brackish water or seawater^29,32–34^.

The great ability of *P. khori* to tolerate a wide range of salinities is directly linked to its evolved osmoregulatory power, which enables the maintenance of haemolymph osmolality within a stable range, providing osmotic protection to the cells^47,48^. The results of muscle water content illustrate the protection provided by the remarkable osmoregulatory ability of the shrimps. It was observed that stability in muscle water content occurred at salinities where the shrimps were actively engaged in hypo-hyper-osmoregulation. A decrease in muscle water content was only observed when the shrimps transitioned to a hypo-conformation pattern. This transition led to an increase in haemolymph osmolality, thereby imposing an osmotic challenge on the cells. The osmotic challenge may have intensified at the higher experimental salinity, reaching a limit of tolerance and resulting in death.

This study demonstrated that *P. khori* exhibits the strongest recorded osmoregulation capacity among palaemonid shrimps and one of the strongest osmolality capacities among carideans, tolerating salinity levels from 18 to 68, with >  60 to 68 as the highest salinity level tolerated. In this study, *P. khori* exhibited a haemolymph osmolality close to 1000 mOsm/kg H_2_O at a modal salinity of approximately 40 ppt, with haemolymph isosmotic points above 900 mOsm/kg H_2_O. Notably, *P. khori* sustained a haemolymph osmolality 560 mOsm/kg H_2_O below the osmolality of salinity 68 ppt, indicating an extraordinary osmotic capacity. This is an unprecedented finding, as there are no previous records in the literature of a caridean shrimp exhibiting such hypo-osmoregulation in such high salinity environments^24–27^. For comparison, other palaemonid shrimps such as *P. serratus* (Pennant, 1777), *P. affinis* H. Milne Edwards, 1837, *P. macrodactylus* Rathbun, 1902, *P. varians* Leach, 1814, and *P. northropi* (Rankin, 1898) typically exhibit haemolymph osmolality between 600 and 770 mOsm/kg H_2_O at modal salinities between 20 and 25, with haemolymph isosmotic points between 566 and 685 mOsm/kg H_2_O^46^. Although *P. northropi* can tolerate higher salinity levels, its regulation efficiency diminishes at salinities above 35 ppt^29^. Similarly, *P. affinis* demonstrates hyper-osmoregulation in salinities between 5 and 20 ppt and hypo-osmoregulation in 35 to 43 ppt, with low survival rates in salinities above 35 ppt^49^. Another study revealed that *P. northropi* survives remarkably well in media ranging from 1.5 to 45 salinity, and for up to 24 h in salinity of 50, much like *P. affinis, P. longirostris* H. Milne Edwards, 1837 and *P. pandaliformis* (Stimpson, 1871)^46^. While *P. khori* surpassed these capabilities by surviving more than 24 h in a salinity of 68 ppt and with 80% of specimens surviving in a salinity of 63 ppt for up to 48 h. Among carideans, only the Hawaiian anchialine shrimp *Halocaridina rubra* Holthuis 1963, demonstrates comparable osmoregulation capacity, tolerating salinities ranging from 0 to 56 ppt and although it sustains a gradient of 868 mOsm/kg H_2_O in freshwater, however, at salinity 56 the osmotic gradient sustained was not as pronounced (264 mOsm/kg H_2_O below external salinity)^27,50^. It is worth noting that further studies are needed to determine the lowest salinity level tolerated by *P. khori* because in this study the experiment started with 18 ppt as the lower salinity level. These findings underscore the efficient physiological adaptation of *P. khori* to hyperarid mangrove environments, suggesting a close relationship with the brine groundwater recorded in these habitats. Therefore, the osmoregulatory capacity of *P. khori* likely represents a habitat-related evolutionary adaptation, with optimal osmoregulation linked to the natural salinity of their environment. This adaptation may have evolved from marine ancestries inhabiting estuaries or tidal creeks on a seasonal basis, as suggested in the literature about decapod osmolality adaptations^27,50^.

The phylogenetic results demonstrated the existence of different congeners within the *P. debilis* complex, with several species still taxonomically unclassified. This study also confirms the genetic similarity of *P. khori* with representatives of the *P. debilis* complex, such as previously described morphologically^16^. The *P.* aff*. khori* recorded in the present study is related to *P. khori* and genetically different from other congeners of the *P. debilis* complex^34,51^. This palaemonid recorded in the intertidal rocky shore in Shamal, Qatar, remains taxonomically undescribed.

The speciation of palaemonid species is recorded with different congeners inhabiting specific areas and niches according to salinity levels^29,32–34^, as discussed before. In this study, we recorded *P. khori* in dense biomasses dominating the niche with high salinity, and *P.* aff. *khori* inhabiting the intertidal zone in the marine ecosystem. Suggesting a niche division, with speciation possibly related to the salinity of the inhabited ecosystem. In addition, the speciation of these two species, apart from the other congeners from the *P. debilis* complex, is possibly related to the salinity recorded in the southeastern portion of the Arabian Gulf^5^, a marine region with a geological history marked by the existence of saline lakes and recorded as responsible for the speciation of invertebrate species^51–53^. The dietary relationship with the mysid from salt pans, the osmoregulatory power and the recorded high salinity of the groundwater suggest that the speciation of *P. khori* is related to the groundwater in the studied arid mangrove complex.

From a phylogeographic point of view, *P. khori* and *P.* aff. *khori* are two recorded representatives of *P. debilis* complex that inhabits the Southwestern Arabian Gulf ecoregion. The confirmation and identification of the representative of *P. debilis* complex from the Red Sea^21^ remains unclear. Beyond Hawaii on the Pacific Ocean, the type locality of *P. debilis*, the other representatives of this complex also require confirmation. The absence of *P. khori* in other mangroves in the Gulf (Reza Naderloo *per.com*.)^20–23^, including its absence in other mangroves in Qatar as recorded here, illustrates the endemism level of this species. From an ecosystem preservation point of view, this biogeographic isolation of *P. khori* in a single mangrove setting highlights the importance of preserving this hyperarid mangrove ecosystem.

The time tree analysis demonstrated that the *P. debilis* complex, reported in taxonomic references^16,21^, emerged in the Eocene epoch between 53 and 42 MYA and was possibly related to the Paleocene–Eocene Thermal Maximum (PETM), the main event recorded in the late Paleocene early Eocene and responsible for a mass extinction on earth due the temperature rise of and related climate events, including raise of sea level on PETM and sequential level decrease during the Eocene^54^. Highlighting that in the Paleocene in the early Cenozoic, the foreland Persian basin was already formed after the collision between the Arabian and the Eurasian plates^55^, and therefore, before the speciation of *P. khori* and *P.* aff. *khori.* Suggesting that the speciation of the studied species could have occurred within the Arabian Gulf.

Correlating the evolutionary history of the studied *Palaemon* with its habitat, evidence suggests that the presence of mangroves and associated biodiversity first emerged during the Late Cretaceous period along the coasts of the Tethys Sea^56–58^. However, new evidence continues to emerge regarding the mangrove’s origin. Records of mangroves in the region around the Arabian Gulf were found in the Eocene period. Fossil evidence of mangroves has been discovered in formations such as the Eocene Rusayl Shale Formation in Oman during the Middle-Late Eocene, in Western Anatolia, Turkey, and includes the presence of *Avicennia* fossils, the only mangrove tree in the studied ecosystem, that has also been documented in the lower Eocene of the Mediterranean^59–62^. Importantly, it is worth noting that the first geological record of mangrove fossils in the Arabian Gulf also dates back to the Eocene period, the same period when the studied *Palaemon* emerged, including findings in the Qom Formation in Iran^63^ and the Dammam Formation in Qatar^64^. The speciation of *P. khori* coincides with the geological period when the mangrove ecosystem was first recorded in Qatar, including the presence of similar biodiversity associations in both fossilised and modern mangrove populations^64^. These findings establish a strong link between the evolutionary history of *P. khori* and the studied ancient Athalassic mangrove oasis environment.

## Methods

### Studied area

The studied area is the Al-Khor & Al-Dakhyra mangrove complex, the large hyperarid Mangrove settings of Qatar, in the southwestern ecoregion in the Arabian Gulf (Fig. 1A). With the absence of rivers, this mangrove is marked by the existence of tide channels forming the riparian zone (Fig. 1B), similar to riparian zones formed in the traditional mangroves on the border of rivers, but here, physically formed by the daily tide oscillation. The channels open in bays and are connected with beaches in the coastal areas. Two small fragmented mangroves were also visited for the qualitative benthonic surveys, including the natural deforested mangroves in Shamal, located and less than 50 km north of the Al-Khor & Al-Dakhyra mangrove complex and the planted mangrove setting in Al-Wakrah^65^ around 50 km south of the same mangrove complex (Fig. 1A).

### Biological samplings

Aiming to search for *P. khori* in the mangroves in Qatar, snorkelling samplings through visual identification was performed. Dives were performed during low tides in the channels of the Al-Khor & Al-Dakhyra mangrove complex, in the mangrove fragments on the Shamal coast (north of Qatar) and in the planted mangrove in Al-Wakrah (southeast of Qatar). Considering the natural, expected habitat of *P. khori*, the snorkelling dives were focused on the riparian zone (Fig. 1B). Representatives of *P. khori* observed in the studied mangroves were manually collected using hand plankton nets for genetic studies. For the salinity tolerance experiments, representatives of *P. khori* were collected from a shrimp biomass in the riparian zone at the Al-Khor & Al-Dakhyra mangrove complex (Fig. 1A,B). Specimens were taken for taxonomic identification^16^.

### Abiotic samplings

Considering the literature review, the studied hyperarid mangrove ecosystem exists without a freshwater input^5,17^. However, in this study, we hypostatise that brackish groundwater water with low salinity might influence the origin and existence of this ecosystem by considering the salinity oscillation recorded in previous studies^5,17^ and the existence of groundwater-dependent oasis ecosystems^66^, including the existence of mangroves linked to groundwater existence^36^.

By considering the high temperature previously recorded during summertime in the shallow water environment in Qatar, trespassing 40 °C^5,17^ and the expected “colder” temperature naturally observed in groundwater outflow, it is possible to assume that in a warm water environment, cold water outputs on the ground of mangrove channels will indicate the presence of groundwater outflow. Therefore, the methodology is based on actively walking in the channels and tidepools in the studied Mangrove setting, during the lower tides, in the summertime, and searching for cold water outflow. Temperature and salinity were measured in the mangrove channels using the Probe AK 88 multiparameter^®^. When a cold groundwater water output was spotted, temperature and salinity were measured using the same probe, and some water samples were collected. The 50 ml falcon tubes were immersed and still sealed in the mangrove channel, and it was just opened in the exact groundwater outflow spot to avoid/reduce the contamination with the mangrove water. Salinity was subsequently determined at the QU Marine Lab using a Salinity refractometer (with an Automatic Temperature Compensation, Portable Refractometer).

### Trophic chain

Aiming to understand the diet and the related trophic chain of *P. khori* and considering their recorded carnivorous diet^17^ planktonic and macro specimens were collected in the riparian zone next to the shoals of *P. khori*, using plankton hand-nets with 300 µm aperture. A complimentary material was collected on the surface of the submerged root in the riparian zone for comparison, mostly covered by red algae. The isotope analysis focused on carbon (δ^13^C) and nitrogen (δ^15^N) to identify the origins and level of the components in the trophic chain of *P. khori*. The plankton samples were categorised by species using a stereomicroscope (Olympus CX22LED^®^). Each isolated category was dehydrated for 48 h in an incubator oven at 45 °C with a fan set at 90% (Memmert^®^). The material was analysed at the Laboratório de Ecologia Isotópica, Centro de Energia Nuclear na Agricultura—CENA, Universidade de São Paulo—USP. Stable isotope ratios in the samples are expressed as delta notation (δ, ‰).

### Osmolarity

In the Laboratory, the collected shrimps (Fig. 3A,B) were placed in an aquarium containing natural mangrove water with a salinity of 42 ppt and constant aeration. The shrimps were allowed to acclimate for 2 days, under a natural photoperiod, before being transferred to the experimental conditions. A series of aquariums was set up using marine salt and distilled water to achieve the following salinities: 18, 28, 38, 43, 48, 53, 58, 63, 68, 78 (ppt). More specifically, 3 aquariums were prepared for each salinity, resulting in a total of 30 aquariums (Fig. 3C,D). Salinity measurements were conducted using the same probe employed in the field.

Considering the high density of the shrimp biomass in nature (Fig. 3A), the number of specimens per aquarium was not considered a concern. A minimum of 10 shrimps were placed per aquarium. In the case of small specimens, additional individuals were added to reach a pool of shrimps at the end of the experiment and ensure a measurable volume of haemolymph. During the salinity exposure period of 3 days, the shrimps were not fed, and the water was renewed. Mortality was checked and recorded at 6, 24, 48 and 72 h. After 72 h of exposure, all remaining animals were removed from the tanks and the haemolymph was extracted by cardiac puncture using a micropipette. For small specimens, when the volume of haemolymph didn’t reach 2 μL we did the pool of haemolymph using more animals from the same aquarium (due to the specificity of the osmometer). Haemolymph osmolality was read using the osmometer (micro-osmometer Vapro 5600, Wescor, USA) using 2 μL of undiluted samples of *P. khori* specimens exposed to different salinities. Unities are mOsmol/kg H_2_O.

Tissue hydration analysis was based on the muscle water content comparing the wet weight and the dry weight of all specimens that survived the 72 h of exposure to each treatment. Muscle samples were removed from the abdominal muscle and gently blotted dry with a paper filter and the wet weight was recorded in a precision balance (Mettler Toledo/XS403S). The muscle samples were then dried at 60 °C for 24 h in an oven (Memmert^®^), and then weighed again using the same analytical balance used for the wet weight.

The Mortality, Muscle Water Content and Osmolarity were evaluated to compare the osmotic strategy and the salinity level’s tolerance for this endemic species. Data are presented as mean  ±  standard error of the mean and were submitted to statistical analysis. Data showed non-normal distribution and were compared using Kruskal–Wallis one-way ANOVA on Ranks and Dunn’s Method was used as post-hoc test. Charts were prepared to illustrate the results.

### Phylogeny and phylogeography

The genetic study of *P. khori* aims to understand the phylogeny and the evolutionary aspects related to its restriction on the mangrove at Qatar, a very abundant but secluded biomass. We aim to compare also the genetic similarity among the specimens from Al-Khor & Al-Dakhyra mangrove complex, its original type locality of this species it type^16^, with the species from the mangroves at Shamal and Al-Wakrah.

The collected shrimps were forwarded to BRC facility conserved in 70% ethanol. Around 200 mg of the abdomen specimen were cut, washed three times in PBS buffer at 4000×*g* for 5 min, homogenised in G2 lysis buffer and subjected to DNA extraction with the modified protocol of the Genomic Tips 100G Kit (Qiagen) as described elsewhere^67^. The quality of extracted DNA was evaluated using the A260/A280 ratio by Nanodrop as well as by agarose gel electrophoresis. The sample quantification was achieved using the Qubit^®^ dsDNA High Sensitivity Assay Kit (ThermoFisher Scientific).

The extracted DNAs were subjected to PCR amplification targeting two genes: 16S and COI genes using 2X HotStart Taq *plus* master mix (Qiagen) in a total reactional volume of 25 µl. The large subunit rRNA 16S gene was amplified using the forward primer 1472 (5′-AGATAGAAACCAACCTGG-3′) and the reverse primer 16L2 (5′-TGCCTGTTTATCAAAAACAT-3′) according to the cycling conditions of initial denaturation 5 min at 95 °C followed 30 cycles of 20 s at 95 °C, 20 s at 48 °C and 45 s at 72 °C for as well as a final extension step for 5 min at 72 °C^35^. The universal metazoan primers set LCO1490 (5′-GGTCAACAAATCATAAAGATATTGG-3′) and HCO2198 (5′-TAAACTTCAGGGTGACCAAAAAATCA-3′) were used for the amplification of the cytochrome C oxidase subunit 1 gene after 35 cycles of 1 min at 95 °C, 1 min at 40 °C, 90 s at 72 °C and a final extension step at 72 °C for 7 min as reported elsewhere^68^. The endpoint products of the different amplification reactions were then subjected to 1.2% agarose gel electrophoresis visualisation and purification step using the Monarch^®^ PCR & DNA Cleanup Kit (NEB). The clean products were sent for direct sanger bi-directional sequencing at Macrogen Inc., South Korea. The generated DNA sequences resulting from this study were deposited in the GenBank with accession numbers presented in the sections below.

The generated sequences were viewed, analysed, edited, primers trimmed with BioEdit software v7.2.5^69^, and then subjected to BLAST-N approach to confirm their identities. Multiple alignments with homologous sequences deposited in the NCBI database were achieved by MAFFT software with G-INS-i strategy^70^. Phylogenetic partitions and optimal substitution models were identified using the Bayesian Information Criterion (BIC) and the Akaike Information Criterion (AIC) metrics as implemented in MEGA X v10.0.5^71^.

The most appropriate models for maximum likelihood analyses were revealed to be HKY  +  G  +  I for both 16S and COI datasets. MrBayes (v3.2.7) was run for each marker under its appropriate model, each with Markov Chain Monte Carlo (MCMC) chain for a minimum of 1.000.000 generations until the average standard deviation STD of the split frequencies reached levels below 0.01. Hence, the final Bayesian analysis was conducted for 1 million generations for the 16S and the COI palaemonid datasets. Trees were sampled every 1000 generations with the first 25% of them discarded as burning and the rest used for the Consensus trees construction. the clade support of the generated tree was evaluated with each node posterior probability values.

Maximum Likelihood phylogenetic trees were constructed for the COI palaemonid dataset, based on 1000 bootstrap replications, with Real-Time ML clock calculation^72^. The choice of suitable time boundaries to identify the divergence time between the species was calculated in accordance with the data extracted from TimeTree resource base^71^.

## Data Availability

All data produced in this study are available in repositories. Genetic data deposited in the Genbank—NCBI (https://www.ncbi.nlm.nih.gov/genbank/): (Accession number Isolate_*P.khori*: OQ421809) by C (Accession number Isolate_*P.*aff. *khori*: OQ421808).
